# Culturing C2C12 myotubes on micromolded gelatin hydrogels accelerates myotube maturation

**DOI:** 10.1186/s13395-019-0203-4

**Published:** 2019-06-07

**Authors:** Lance T. Denes, Lance A. Riley, Joseph R. Mijares, Juan D. Arboleda, Kendra McKee, Karyn A. Esser, Eric T. Wang

**Affiliations:** 1 Department of Molecular Genetics and Microbiology, Center for Neurogenetics, Myology Institute, College of Medicine, University of Florida, Gainesville, FL 32610 USA; 2 Department of Physiology and Functional Genomics, Myology Institute, College of Medicine, University of Florida, Gainesville, FL 32610 USA

**Keywords:** C2C12, RNAseq, Myotubes, Micromolding, Hydrogels, Sarcomere

## Abstract

**Background:**

Skeletal muscle contributes to roughly 40% of lean body mass, and its loss contributes to morbidity and mortality in a variety of pathogenic conditions. Significant insights into muscle function have been made using cultured cells, in particular, the C2C12 myoblast line. However, differentiation of these cells in vitro typically yields immature myotubes relative to skeletal muscles in vivo. While many efforts have attempted to improve the maturity of cultured myotubes, including the use of bioengineered substrates, lack of molecular characterization has precluded their widespread implementation. This study characterizes morphological, molecular, and transcriptional features of C2C12 myotubes cultured on crosslinked, micropatterned gelatin substrates fabricated using previously established methods and compares them to myotubes grown on unpatterned gelatin or traditional plasticware.

**Methods:**

We used immunocytochemistry, SDS-PAGE, and RNAseq to characterize C2C12 myotubes grown on micropatterned gelatin hydrogels, unpatterned gelatin hydrogels, and typical cell culture substrates (i.e., plastic or collagen-coated glass) across a differentiation time course. The ability to form aligned sarcomeres and myofilament protein concentration was assessed. Additionally, the transcriptome was analyzed across the differentiation time course.

**Results:**

C2C12 myotubes grown on micropatterned gelatin hydrogels display an increased ability to form aligned sarcomeres as well as increased contractile protein content relative to myotubes cultured on unpatterned gelatin and plastic. Additionally, genes related to sarcomere formation and in vivo muscle maturation are upregulated in myotubes grown on micropatterned gelatin hydrogels relative to control myotubes.

**Conclusions:**

Our results suggest that growing C2C12 myotubes on micropatterned gelatin hydrogels accelerates sarcomere formation and yields a more fully matured myotube culture. Thus, the use of micropatterned hydrogels is a viable and simple approach to better model skeletal muscle biology in vitro.

**Electronic supplementary material:**

The online version of this article (10.1186/s13395-019-0203-4) contains supplementary material, which is available to authorized users.

## Background

Skeletal muscle accounts for approximately 40% of body mass and is essential for both locomotion and whole body metabolism [1, 2]. Loss of skeletal muscle mass during aging and pathogenesis is a known contributor to morbidity and mortality, thus a large contingent of research aims to prolong health by maintaining muscle quality [3–7]. While there is no replacement for studies of muscle in vivo, muscle cell culture models allow for more rapid and facile manipulation to address mechanistic questions and perform drug screening experiments. Thus, there is a need for in vitro models of skeletal muscle that mimic the in vivo tissue.

The C2 myoblast was developed in 1977 as a control cell line to study muscular dystrophies in vitro [8]. Since then, the C2C12 subclone has become widely used in the skeletal muscle field as a cell culture model [9–12]. When exposed to low-serum differentiation media, these cells differentiate and fuse into a multinucleated syncytium referred to as a myotube. C2C12 myotubes express contractile proteins and, when left to differentiate for an extended period of time, can spontaneously contract. These properties make C2C12 cells an invaluable tool for understanding the molecular biology of muscle development. However, these cells do not perfectly mimic in vivo muscle fibers. The contractile proteins present in these cells are typically disorganized and rarely form aligned sarcomeres, and the biological pathways studied are often unrepresentative of mature muscle [13, 14]. Thus, there is a need to develop a culture system that allows further maturation of myotubes in order to more accurately model in vivo skeletal muscle biology.

Historically, C2C12 myoblasts have been cultured on uncoated cell culture dishes until they near confluence. At this stage, myoblasts are serum withdrawn to induce differentiation and fuse into multinucleated, post-mitotic myotubes [11]. Over the next several days, myotubes develop similarly to embryonic skeletal muscle, but often detach from the cell culture dish after approximately 7 to 10 days due to spontaneous contraction [15, 16]. Because detachment of myotubes leads to cell death and presents obvious challenges for subsequent study, cultured myotubes are generally unsuitable for long-term studies. This problem has been addressed by coating cell culture dishes with substrates such as collagen, gelatin, and Matrigel™ (Corning) that allow enhanced adhesion and/or modulate the stiffness of the surface such that detachment is delayed, but prolonged culture of myotubes on these substrates is still not possible [17, 18]. To address these shortcomings, biomedical engineers have developed methods that permit culture of more mature myotubes in vitro, including bioengineered substrates, 3D culture systems, and paradigms that include electrical stimulation or mechanical stretching [19–25]. Though some methods have been successful, the resultant myotubes have not been sufficiently characterized, particularly at the molecular level. Additionally, technical challenges preclude the implementation of many of these methods in basic biology laboratories. For these reasons, many skeletal muscle labs continue to use suboptimal culture substrates for C2C12 studies.

We set out to characterize molecular and cell biological features of C2C12 myotubes grown using a bioengineered substrate in an effort to provide the skeletal muscle community an easy to use resource for developing more mature myotubes. After a literature review, we chose to use the micropatterned gelatin hydrogel system developed by Bettadapur et al. (2016) due to its ease of implementation in the setting of a basic biology lab, as well as their reports of morphologically advanced and prolonged cultures [26]. Through studying sarcomere morphology, protein expression, and transcriptomics, we found that micropatterned gelatin hydrogels accelerate and advance myogenic maturation and sarcomere formation in C2C12 myotubes when compared to traditional culture methods. This study provides the morphological and molecular information that can be used by investigators to determine whether the benefits of micropatterned culture justify their implementation. In our opinion, the patterning approach used here is cheap, facile, and easily adaptable by most skeletal muscle biology laboratories using C2C12 myotubes as an in vitro model.

## Methods

### Fabrication of PDMS stamps and gelatin hydrogels

PDMS stamps and gelatin hydrogels were fabricated according to the protocol by Bettadapur et al. (2016) [26]. Briefly, silicon wafer templates were made through the University of Florida Nanoscale Research Facility such that the photomask consisted of 10 μm lanes by 10 μm gaps (10 × 10) as 10 μm is roughly the width of a cell. The design file used to produce the photomask is included in Additional file 1. Elastomer base and curing agent from the Sylgard 184 silicone elastomer kit (Dow Corning) were mixed at a 10:1 ratio then poured over the wafer template, degassed, and cured in an oven at 65 °C for 4 h. PDMS stamps were then removed from the wafer and cut to fit on 22 mm × 22 mm glass coverslips.

Ten percent weight/volume gelatin was prepared with Bloom type A porcine gelatin (Sigma, St. Louis, MO) with autoclaved water and dissolved at 65 °C. Ten percent gelatin solution was added dropwise onto cell culture dishes. Sterilized 10 × 10 micropatterned or flat (i.e., unpatterned) PDMS stamps were then pressed onto the gelatin solution and incubated overnight at room temperature. With the stamp still in place, the gelatin was rehydrated with PBS for at least 3 h. PDMS stamps were then carefully removed from the gelatin, and the hydrogel was then incubated in a 10% *w*/*v* microbial transglutaminase solution dissolved in autoclaved water for 4 h at room temperature. Plates were washed with PBS and UV sterilized prior to use.

### Cell culture

The mouse myogenic C2C12 cell line was obtained from ATCC and cells were used up until passage number 8. Myoblasts were maintained on plastic cell culture dishes in Dulbecco’s modified Eagle’s medium (DMEM) supplemented with 10% fetal bovine serum and 1% penicillin-streptomycin in a humidified incubator kept at 37 °C and 5% CO_2_. When cells reached 70% confluency, they were separated and plated on micropatterned gelatin hydrogels, unpatterned gelatin hydrogels, or on a fresh plastic cell culture dish at 1.5 × 10^5^ cells per 35 mm dish. Once confluent, cells were serum restricted with differentiation medium (DMEM, 2% horse serum, 1% penicillin-streptomycin) and left to differentiate for either 4 or 7 days. Growth or differentiation medium was replenished daily.

Skeletal muscle primary myoblasts were isolated as previously described [27, 28]. Hindlimb muscles from 3 8-week-old C57/Bl6J mice were collected and minced in Ham’s F-10 supplemented with 10% fetal bovine serum and 1% penicillin-streptomycin. Minced tissues were then incubated in 800 U/ml type II collagenase (Worthington) at 37 °C for 1 h with gentle agitation. Samples were then triturated with a 20 G needle, centrifuged, and resuspended in wash medium. Cell suspensions were filtered and resuspended in Ham’s F-10 supplemented with 20% fetal bovine serum, 1% penicillin-streptomycin, and 2.5 ng/ml basic fibroblast growth factor. Resuspended cells were pre-plated for 30 min in an uncoated dish, then unattached cells were transferred to ECM-coated (ECM gel from Engelbreth-Holm-Swarm murine sarcoma, Sigma) dishes. When cells reached 70% confluence, they were trypsinized and plated on micropatterned gelatin hydrogels. At this point, they were treated the same as C2C12 cells.

### Imaging

A plasmid containing an eGFP labeled α-actinin-2 (ACTN2-pEGFP) was a gift from Johannes Hell (Addgene plasmid #52669) [29]. The ACTN2-pEGFP gene fusion was then inserted into a PiggyBac Transposon system expression vector using standard molecular cloning techniques to facilitate stable cell line generation by puromycin selection (System Biosciences, Palo Alto, CA). C2C12 myoblasts were co-transfected with the ACTN2-pEGFP expression vector as well as a plasmid containing the Super Piggybac Transposase (System Biosciences, Palo Alto, CA). Following puromycin selection, cells were separated using a flow cytometer to select for the low expressing GFP cells to limit the effect of overexpressing α-actinin-2 for our studies. Cells were maintained as described above; however, to aid in microscopy, cells were grown on micropatterned or unpatterned gelatin-coated glass coverslips activated using 100 mM NaOH, 0.5% (3-Aminopropyl) trimethoxysilane, and 0.5% glutaraldehyde as described in Bettadapur et al. (2016) prior to being fixed with 2% paraformaldehyde. Cells were imaged using the ZEISS LSM 880 confocal microscope in the GFP and DAPI channels to visualize sarcomeric Z-lines and nuclei respectively. Sarcomere lengths were measured using fast Fourier transformations using the SarcOptiM plugin for ImageJ [30]. Specifically, the line tool in ImageJ was used to draw a line across 15 sarcomeres in series. SarcOptiM then computed the FFT spectrum based on the gray level profile along this line by looking for the peak in the defined sarcomere length range (defined by us as 1.6 um to 3.4 um). The number of myotubes forming sarcomeres was determined by counting the total number of myotubes within an image and the number of these myotubes that contained visually discernible sarcomeres. Live cell imaging of myotube contraction (Additional file 5) was performed using the ZEISS LSM 880 microscope outfitted with a ZEISS live cell imaging chamber and CCD camera. Imaging was performed at 37 degrees Celsius and 5% CO2. EGFP α-actinin-2 C2C12 cell lines were differentiated for 7 days on patterned gelatin substrates in glass bottom cell culture dishes. Media was replaced with Gibco FluoroBriteTMmedia supplemented with 2% horse serum for imaging. Images were collected once per second for 100 s.

### Determination of contractile protein content

Protein content of myosin heavy chain and actin were calculated from total protein lysates isolated from C2C12 myotubes grown on plastic dishes, unpatterned gelatin hydrogels, and micropatterned gelatin hydrogels. C2C12 cultures at days 4 and 7 of differentiation were briefly exposed to 0.05% trypsin to enrich for myotubes similar to the protocol used by Bi et al. (2017) [31]. Trypsin was deactivated with an excess of cell growth medium (DMEM, 10% FBS, 1% P-S) then spun at 500x*g* for 3 min at 4 °C. Pellets were washed with PBS then dissolved by directly adding 150 μl extraction buffer containing 2% SDS, 10% glycerol, 50 mm Tris base, 2% 2-mercaptoethanol, pH8.8 to cells grown in a 3.5 cm dish [32]. Protein concentration was measured using the Bio-Rad DC protein assay (Hercules, CA), and samples were diluted to 1 μg/μl with 4x Laemmli buffer before being boiled at 95 °C for 5 min. Equal amounts of total protein (5 μg) were loaded onto a 7.5% SDS-polyacrylamide gel and separated by molecular weight (100 V for 100 min) according to Baummann et al. (2016) [33]. Following electrophoresis, gels were fixed in 50% methanol 7% glacial acetic acid for 30 min then transferred into SYPRO Ruby protein stain (Molecular Probes, Eugene, OR) overnight. Stained gels were washed in 10% methanol, 7% glacial acetic acid for 30 min then with water prior to imaging. Protein gels were imaged using the Bio-Rad ChemiDoc MP and densitometry was performed for myosin heavy chain (~ 220 kDa), actin (~ 42 kDa), and total protein within each lane of the gel with Image Lab software.

### RNA preparation and RNAseq

C2C12 and primary myoblast cultures at days 4 and 7 of differentiation were processed similarly to those described above. Trypsin was deactivated with an excess of cell growth medium (DMEM, 10% FBS, 1% P-S) then spun at 500x*g* for 3 min at 4 °C. Pellets were washed with PBS, dissolved in Trizol, and frozen at − 80 °C until use. RNA isolation was performed using the commercially available Zymo RNA miniprep kit (Zymo Research, Irvine, CA). RNAseq libraries were prepared using the commercially available NEBNext kit (New England Biolabs, Ipswitch, MA). Libraries were quantitated using the Qubit system (Invitrogen, Carlsbad, CA), and fragment size determined using the Applied Biosystems Fragment Analyzer (Foster City, CA). Libraries were pooled and loaded at 20 pM onto an Illumina NextSeq 500 High Output v2 flow cell (75 × 75) (Illumina, San Diego, CA), generating roughly 35 million paired-end reads per sample.

FastQ files were downloaded to the University of Florida HiPerGator computing cluster. Differential gene expression analysis was performed using the programs Kallisto and Sleuth [34, 35]. Gene ontology enrichment analysis was performed using the online Panther GO enrichment tool [36–38]. All downstream analysis was performed using custom scripts in the R and Python languages.

### Statistical analysis

A one-way ANOVA with Tukey’s multiple comparisons was performed to determine significant relationships between groups in IHC and contractile protein content experiments. Statistical significance for these experiments was set a priori at *P* < 0.05 except for RNAseq data where *Q* < 0.05 was considered significant. Data are represented as mean ± SEM. Data analyses and histograms for IHC and contractile protein experiments were conducted with Prism (GraphPad, La Jolla, CA), while RNAseq experiments were analyzed and graphed using custom Python and R scripts. Wilcoxon rank-sum tests were performed to determine significant changes in log_2_(fold change) distributions between various sets of genes in the transcriptomics analysis experiments. Data analysis and statistical testing were performed in Python.

## Results

### Micropatterned C2C12 myotubes develop more sarcomeres than those grown on other substrates

**Fig. 1:**
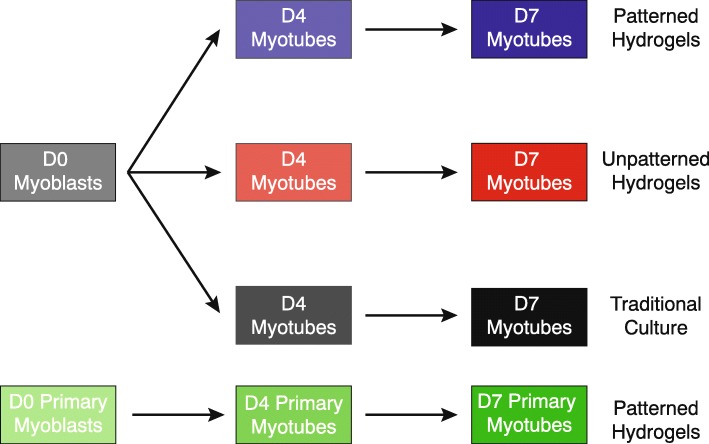
Experimental timecourse of C2C12 and primary myotubes on micropatterned hydrogels, unpatterned hydrogels, or using traditional cell culture. C2C12 cells or primary myoblasts were cultured on either micropatterned gelatin hydrogels, unpatterned gelatin hydrogels, or using traditional models of cell culture (i.e., collagen-coated glass coverslips or plastic cell culture dishes)

In this study, we compared C2C12 myotubes cultured on three different substrates. The first was a micropatterned gelatin hydrogel with 10 μm-wide grooves as previously described (see “Methods” section) [26]. The second was the same gelatin hydrogel but lacking grooves. The third was either plastic (molecular experiments) or collagen-coated glass (morphological experiments) to mimic the predominant cell culture methods used by the skeletal muscle research community. Cells were analyzed at day 4 of differentiation (D4) and day 7 of differentiation (D7) (Fig. 1). Some analyses also used primary myoblasts from C57Bl6/J gastrocnemius [27] as a reference, also differentiated for 4 or 7 days on micropatterned gelatin hydrogels.

**Fig. 2:**
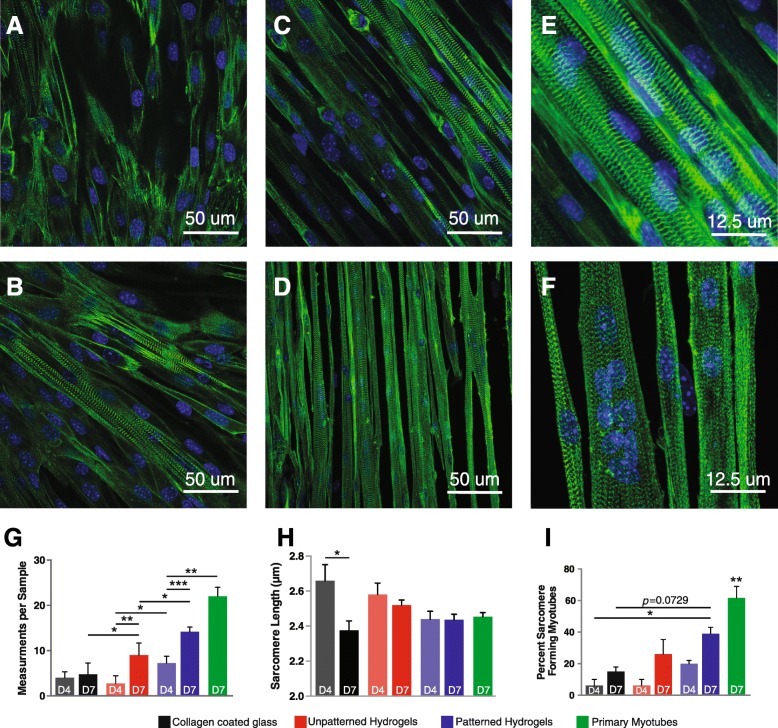
Sarcomere formation is accelerated in C2C12 myotubes grown on micropatterned gelatin hydrogels. Representative images of C2C12 myotubes 7 days post-differentiation grown on **a** collagen-coated glass coverslips, **b** unpatterned gelatin hydrogels, and **c**, **e** patterned gelatin hydrogels, as well as **d** and **f**. primary myotubes grown on patterned gelatin hydrogels. Myotubes are labeled with GFP-sarcomeric-α-actinin (green) and DAPI (blue). Images were analyzed by measuring sarcomere formation as defined by greater than 15 sarcomeres in series. **g** C2C12 myotubes and primary myotubes develop a greater number of sarcomeres in series when grown on micropatterned gelatin than on either unpatterned gelatin or collagen-coated glass. **f** Sarcomere length is stable by day 4 post-differentiation in micropatterned C2C12 and primary myotubes but continues to change in both collagen-coated glass and unpatterned gelatin conditions. **f** Images were visually assessed for the approximate number of sarcomere forming myotubes. C2C12 myotubes grown on micropatterned gelatin hydrogels develop sarcomeres more often, even when there are not enough sarcomeres in series to be included in other measurements. Primary myotubes grown on micropatterned gelatin hydrogels develop significantly more sarcomeres than all other conditions. *P < 0.05. **P < 0.01. ***P < 0.001

Because one defining property of skeletal muscle in vivo is the presence of sarcomeres yet most C2C12 myotube cultures do not yield robust sarcomeres, we examined sarcomere formation in vitro. To facilitate these studies, we generated a stable C2C12 cell line expressing eGFP-tagged α-actinin-2 (ACTN2). Cells were FACS sorted to select low expressors, to mitigate potential unwanted effects of high α-actinin-2 overexpression. Representative images of eGFP-ACTN2 C2C12 cells at D7 on each substrate are shown (Fig. 2a–c, e). Primary myotubes at D7 on patterned gelatin hydrogels served as a reference (Fig. 2d, f). The proportion of myotubes showing 15 or more sarcomeres in series, our cutoff for measuring sarcomere length, was greater at D7 as compared to D4 when cultured on gelatin. Interestingly, cells differentiated on micropatterned gelatin showed greater numbers of myotubes with contiguous sarcomeres as compared to cells differentiated on unpatterned gelatin and on collagen-coated glass (Fig. 2g). We also measured the distance between Z-lines as a measure of sarcomere length. We found that while average sarcomere length was not significantly different between myotubes cultured on each substrate, sarcomere length was stable over time between D4 and D7 when grown on gelatin, in contrast to sarcomere lengths from myotubes grown on collagen-coated glass (Fig. 2h). The sarcomere lengths found in patterned C2C12 myotubes at both D4 and D7 exhibited lengths consistent with the in vivo plateau of the length-tension relationship (approximately 2.4 μm) [39].. Additionally, we visually estimated the total number of myotubes with aligned sarcomeres and found that patterned C2C12s formed significantly more sarcomeres as compared to myotubes cultured on other substrates at D4 (*P* < 0.05; Fig. 2i). These results suggest that micropatterned gelatin hydrogels may promote structural maturation of C2C12 myotubes.

### Contractile protein content is increased in micropatterned C2C12 myotubes

**Fig. 3:**
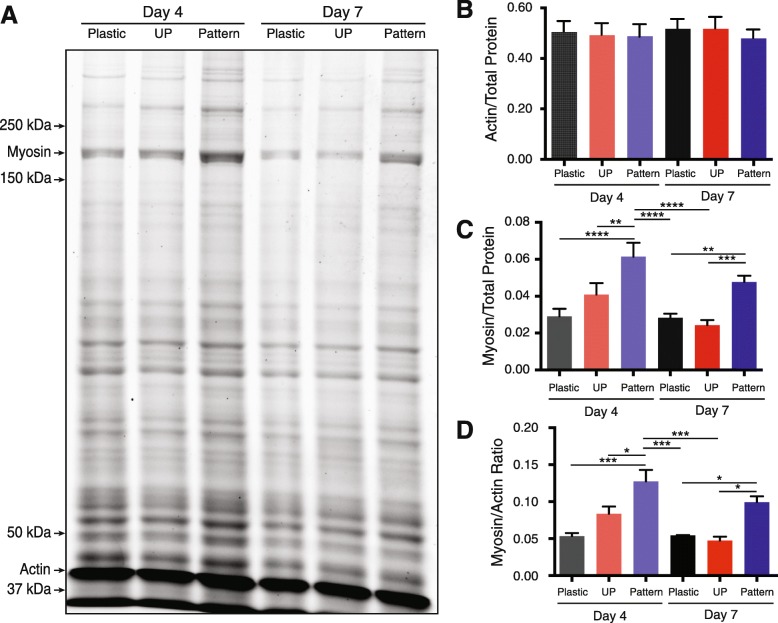
Contractile protein content increases in C2C12 myotubes grown on micropatterned gelatin hydrogels compared to other culturing methods. **a**. Representative SDS-PAGE gel separating total protein content of C2C12 myotubes at days 4 and 7 post-differentiation on plastic cell culture dishes, unpatterned gelatin hydrogels (UP), and micropatterned gelatin hydrogels. **b** Myosin heavy chain content normalized to total protein loaded. **c** Actin compared to total protein loaded. **d** Myosin heavy chain to actin ratio. Sample size per group, *n* = 3 biological replicates. *P < 0.05, **P < 0.01, ***P < 0.001, ****P < 0.0001

We observed that micropatterned myotubes seemed to contract more frequently and uniformly than cells grown on unpatterned gelatin or collagen-coated coverslips. Based on these observations and our sarcomere results, we tested whether micropatterned C2C12 myotubes also produced more contractile protein relative to myotubes differentiated using other methods. We used SYPRO Ruby to stain SDS-polyacrylamide gels loaded with equal amounts of total protein (Fig. 3a). C2C12 myotubes were differentiated on patterned and unpatterned gelatin and compared to myotubes differentiated on plastic. Actin protein content of the myotube culture was not significantly different across any conditions (Fig. 3b). However, we found that the micropatterned myotubes contained significantly more myosin heavy chain and exhibited significantly higher myosin heavy chain versus actin ratios at D4 and D7 relative to the unpatterned control group (Fig. 3c, d). These results demonstrate that micropatterned gelatin hydrogels promote increased contractile protein expression in C2C12 myotubes as compared to other culturing methods. Given our previous observations regarding increased numbers of sarcomeres, the additional myosin heavy chain protein is likely organized into contraction-capable sarcomeres.

### Transcriptomics analysis reveals patterned hydrogel-associated upregulation of sarcomere genes and genes upregulated in vivo

**Fig. 4:**
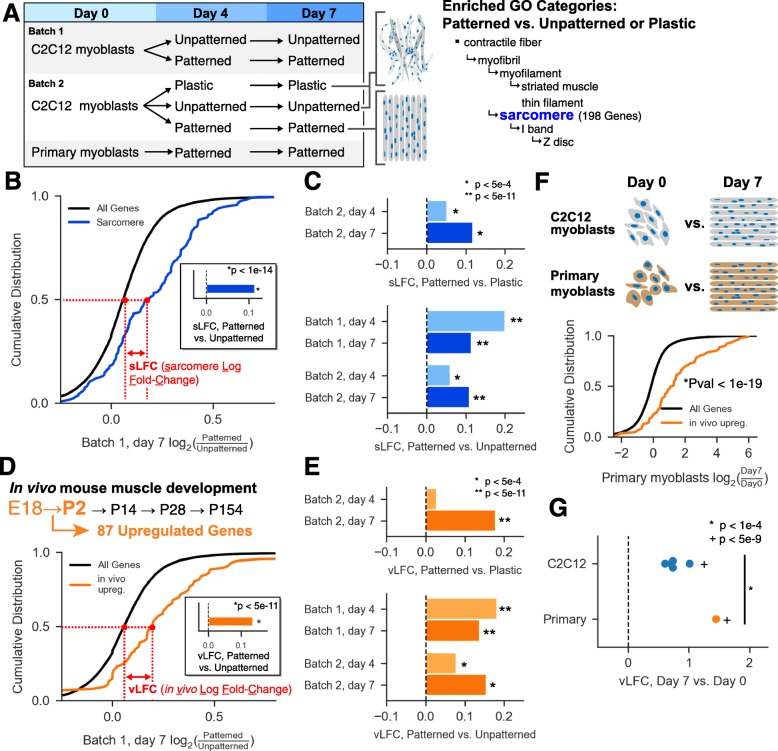
Global transcriptomic characterization of patterned and unpatterned myotubes by RNAseq. **a** Schematic describing samples used for RNAseq. GO categories enriched in all comparisons of patterned vs. unpatterned or plastic myotubes. Genes in the sarcomere category (highlighted in blue) were used for downstream analysis. **b** Cumulative distribution of the log_2_ fold change (LFC) of all genes (black) and sarcomere genes (blue), where LFC is computed using batch 1, day 7 patterned vs. unpatterned myotubes. Inset: bar showing the difference between median sarcomere LFC and median LFC for all genes, defined as sLFC (sarcomere log fold change). **c** Bars showing sLFC for each comparison of patterned vs. unpatterned or plastic. **d** In vivo time course used to select P2 vs. E18 upregulated genes [40]. Cumulative distribution of the LFC of all genes (black) and in vivo upregulated genes (orange), where LFC is computed using batch 1, day 7 patterned vs. unpatterned myotubes. Inset: bar showing the difference between median in vivo upregulated LFC and median LFC for all genes, defined as vLFC (in vivo log fold change). **e** Bars showing vLFC for each comparison of patterned vs. unpatterned or plastic. **f** Cumulative distribution of LFC for all genes (black) and in vivo upregulated genes (orange), where LFC is computed using primary myoblasts at day 7 vs. day 0. **g** Points showing vLFC for all day 7 vs. day 0 myoblast comparisons (blue) and primary myoblasts (orange). The statistical significance of the primary myotube versus C2C12 myotubes was assessed by computing a *Z* score and associated *P* value from the mean and standard deviation of the C2C12 myotubes

To better understand the molecular basis of enhanced sarcomere formation in gelatin-micropatterned C2C12s, we performed RNAseq across the C2C12 differentiation time course. We compared myotubes from two different batches of C2C12 cells (both derived from ATCC stocks) grown on patterned gelatin, unpatterned gelatin, and plastic substrates at day 0 (myoblasts at confluency, just prior to serum withdrawal), day 4, and day 7 (Fig. 4a). Three separate cultures were prepared for each of the 15 conditions, yielding 45 RNAseq libraries in total (all gene expression values are in Additional file 2). Many genes were differentially regulated across time points and growth substrates, but the greatest differences were observed when comparing myotubes grown on plastic versus either gelatin substrate. The number of genes differentially regulated between patterned and unpatterned myotubes was greatest at day 4 (hundreds of genes), and fewer at day 7 (< 100 genes in either batch 1 or batch 2) (Additional file 3). Genes regulated between patterned versus unpatterned conditions were enriched in Gene Ontology categories related to muscle development, and genes regulated between gelatin versus plastic conditions were enriched in categories related to mitochondrial function and cellular respiration (Additional file 4). Interestingly, although we found that specific genes regulated in batch 1 time courses were often not the same genes regulated in the batch 2 time courses, enriched GO categories were remarkably consistent between the two batches. In particular, the “sarcomere” category was overrepresented in all comparisons (Fig. 4a), and therefore we decided to more closely examine the behavior of these 198 genes across each time course. First, we plotted the cumulative distribution of the log_2_ fold change (LFC) between day 7 patterned versus unpatterned samples for all genes and for genes in the “sarcomere” category (Fig. 4b). We then computed the difference in median LFC between those two groups, defined as sLFC (sarcomere log fold change, Fig. 4b). In this comparison, we observed that sarcomere-encoding genes tend to be up-regulated relative to all other genes, yielding an sLFC of ~ 0.1 (Fig. 4b, inset). We extended this approach to all other comparisons of patterned, unpatterned, and plastic myotubes, and observed that all comparisons at days 4 and 7 exhibited significant rightward shifts in sLFC (Wilcoxon rank-sum test), suggesting that patterned substrates broadly increase the expression of sarcomere-encoding genes (Fig. 4c).

We then assessed whether C2C12 differentiation on patterned hydrogels was more representative of in vivo muscle development, relative to differentiation on unpatterned gelatin or plastic substrates. We analyzed a publicly available mouse muscle development RNAseq time course [40], focusing on postnatal day 2 (P2) versus embryonic day 18 (E18) to most closely reflect timing of myoblast to myotube conversion. We identified 143 upregulated and 78 downregulated genes; among the upregulated genes, 87 were expressed in our C2C12 cells. Similar to Fig. 4b, we plotted the cumulative distribution of LFC between patterned and unpatterned conditions, this time separating genes into “*in vivo* upregulated” genes and all other genes (Fig. 4d). We computed the difference in median LFC between those two groups, defined as vLFC (in vivo log fold change), and observed a significant rightward shift when analyzing patterned versus unpatterned C2C12s at day 7 (Fig. 4d, inset). Almost all other comparisons of patterned vs. unpatterned or plastic myotubes also showed a significant rightward shift in vLFC (Wilcoxon rank-sum test), indicating broad upregulation of in vivo upregulated genes upon growth on patterned substrates (Fig. 4e). These analyses suggest that myotube culture on patterned substrates can drive gene expression towards profiles more closely mimicking in vivo muscle development.

Similar to comparisons made in Fig. 2, we sought to compare myotube formation in both C2C12 myoblasts and primary myoblasts on patterned substrates. Here, we plotted the cumulative distribution of LFC between day 7 and day 0, and again separated genes into those upregulated in vivo between E18 and P2 and all other genes (Fig. 4f). We computed the vLFC, yielding ~ 1.4 for primary myotubes at day 7. We also computed similar vLFC values for all C2C12 samples and observed that while all C2C12 samples showed significant rightward shifts, the primary myotubes exhibited the largest shift (Fig. 4g). These results indicate that relative to C2C12 myotubes, primary myotubes cultured on patterned substrates exhibit gene expression changes most closely resembling those occurring in vivo.

## Discussion

In this study, we compared C2C12 myotube maturation on micropatterned and unpatterned gelatin hydrogels to cell culture methods commonly used in the skeletal muscle field. Typically, C2C12 myotubes can be cultured for only 1 week before they detach, and rarely develop structural features characteristic of in vivo muscle fibers [13–16]. By imaging sarcomeric structure, quantitating sarcomeric protein abundance, and characterizing transcriptomes, we show that micropatterned C2C12 myotubes exhibit accelerated maturation relative to unpatterned controls. Our results, combined with previous work in the literature, provide methodology and rationale to implement better cell culturing methods for modeling skeletal muscle in vitro [26]. Further studies will be required to directly compare these in vitro models to in vivo mouse skeletal muscle.

In vitro models reliably modeling in vivo skeletal muscle have been limited due to the time required to differentiate cells to a matured state and the inability to culture myotubes for sufficiently long periods of time. Recently, labs have developed models that prolong C2C12 myotube viability upwards of 21 days post-differentiation [26, 41]. These methods have not been widely adopted by muscle biology labs perhaps because there are technical challenges such as implementing engineering techniques in biology labs (e.g., cost and specialized equipment) and because it is unclear whether the degree to which cultures obtained with these methods exhibit enhanced maturation at a molecular and cellular level. In this study, we found that micromolded gelatin hydrogels provide a substrate that improves myotube maturation based on morphological and molecular indicators. These markers for advanced maturation were seen as early as day 4 of differentiation, suggesting that patterned myotubes not only reach a more mature state but also exhibit accelerated maturation kinetics. Micropatterned C2C12 myotubes increase expression of genes related to sarcomere formation and organization, categories enriched in adult skeletal muscle relative to traditionally cultured C2C12 myotubes [13, 42]. Interestingly, while culturing two different batches of C2C12s on patterns yielded only partially overlapping sets of upregulated genes, both batches showed strong enrichment for sarcomeric genes. This observation highlights the inherent batch to batch variability of differentiated C2C12 myotube cultures at the level of individual gene expression, emphasizing the importance of biological replicates as well as utility of transcriptomic analyses at the network level.

There are a number of reasons why micropatterned gelatin might facilitate accelerated and advanced myogenic development of C2C12 myotubes. Gelatin is naturally derived from collagen, and thus provides a molecular environment more conducive to myoblast/myotube adhesion due to its similarity to extracellular matrix. Additionally, Bettadapur et al. (2016) showed that the elastic modulus of hydrogels is more similar to that of muscle tissue than plastic and other developed substrates (i.e., PDMS) [26]. The combination of a more adhesive and more flexible anchor point against which myotubes can contract reduces the likelihood that they detach from the plate. These effects, due primarily to the molecular composition of the substrate itself, would be present in both patterned and unpatterned gelatin cultures. What then is the underlying biology leading to faster maturation kinetics seen in patterned gelatin substrates? A potential explanation is that patterned scaffolding promotes adherent myoblasts to fuse in only one direction, within a single trough of the hydrogel. The pattern polarizes myoblasts along the axis of the troughs, thus aligning fusion events along a single axis. The myotubes formed are thus less likely to be branched and are longer than those observed in unpatterned cultures. It is likely that reduction in branch points contributes to advanced and prolonged maturation seen, as branching is typically not observed in vivo.

In conclusion, we provide a molecular and cellular characterization of myotubes grown on micromolded hydrogels, and attest that the micromolds are easily implemented by labs with no previous expertise in biomedical manufacturing techniques. We hope our study will encourage widespread adoption of this method in skeletal muscle laboratories making use of C2C12 myotubes as a model system.

## Additional files

Additional file 1: CAD file for generating silicon wafer. (GDS 318 kb)

Additional file 2: Transcripts per million (TPM) for all genes and all analyzed samples. Gene list for the sarcomere GO category and up- and downregulated E18 to P2 in vivo mouse muscle development genes. (XLSX 10615 kb)

Additional file 3: Differential expression analysis output from sleuth for all patterned vs. unpatterned or plastic myotube comparisons. (XLSX 15029 kb)

Additional file 4: Gene ontology enrichment tables from panther for differentially expressed genes in each comparison of patterned vs. unpatterned or plastic myotubes. (XLSX 41 kb)

Additional file 5: Live cell imaging video of spontaneous contractions in eGFP-a-Actn2 C2C12 myotube on patterned gelatin substrate. (GIF 29121 kb)

## Data Availability

RNAseq data has been deposited to GEO (accession numbers pending).

## Abbreviations

ACTN2: α-Actinin 2
GO: Gene ontology
LFC: Log_2_(fold change)
MHC: Myosin heavy chain
PDMS: Polydimethylsiloxane
PSI: Percent spliced in
sLFC: Sarcomere log_2_(fold change)
vLFC: In vivo log_2_(fold change)

## References

[1] Hoppeler H, Flück M (2002). Normal mammalian skeletal muscle and its phenotypic plasticity. J Exp Biol.

[2] Zurlo F, Larson K, Bogardus C, Ravussin E (1990). Skeletal muscle metabolism is a major determinant of resting energy expenditure. J Clin Invest.

[3] Park SW, Goodpaster BH, Strotmeyer ES, de Rekeneire N, Harris TB, Schwartz AV, Tylavsky FA, Newman AB (2006). Decreased muscle strength and quality in older adults with type 2 diabetes. Diabetes.

[4] POWERS SK, LYNCH GS, MURPHY KT, REID MB, ZIJDEWIND I (2016). Disease-induced skeletal muscle atrophy and fatigue. Med Sci Sports Exerc.

[5] Cawthon PM, Fox KM, Gandra SR, Delmonico MJ, Chiou C-F, Anthony MS, Sewall A, Goodpaster B, Satterfield S, Cummings SR, Harris TB (2009). Do muscle mass, muscle density, strength, and physical function similarly influence risk of hospitalization in older adults?. J Am Geriatr Soc.

[6] Wolfe RR (2006). The underappreciated role of muscle in health and disease. Am J Clin Nutr.

[7] Mathur S (2016). Uncovering the factors associated with skeletal muscle weakness in interstitial lung disease. Respirology.

[8] Yaffe D, Saxel O (1977). Serial passaging and differentiation of myogenic cells isolated from dystrophic mouse muscle. Nature.

[9] McMahon DK, Anderson PA, Nassar R, Bunting JB, Saba Z, Oakeley AE, Malouf NN (1994). C2C12 cells: biophysical, biochemical, and immunocytochemical properties. Am J Phys Cell Phys.

[10] Burattini S, Ferri P, Battistelli M, Curci R, Luchetti F, Falcieri E (2004). C2C12 murine myoblasts as a model of skeletal muscle development: Morpho-functional characterization. Eur J Histochem.

[11] Blau HM, Chiu C-P, Webster C (1983). Cytoplasmic activation of human nuclear genes in stable heterocaryons. Cell.

[12] Ikeda K, Ito A, Imada R, Sato M, Kawabe Y, Kamihira M (2017). In vitro drug testing based on contractile activity of C2C12 cells in an epigenetic drug model. Sci Rep.

[13] Deshmukh AS, Murgia M, Nagaraj N, Treebak JT, Cox J, Mann M (2015). Deep proteomics of mouse skeletal muscle enables quantitation of protein isoforms, metabolic pathways, and transcription factors. Mol Cell Proteomics.

[14] Hosseini V, Ahadian S, Ostrovidov S, Camci-Unal G, Chen S, Kaji H, Ramalingam M, Khademhosseini A (2012). Engineered contractile skeletal muscle tissue on a microgrooved methacrylated gelatin substrate. Tissue Eng Part A.

[15] Huang NF, Patel S, Thakar RG, Wu J, Hsiao BS, Chu B, Lee RJ, Li S (2006). Myotube assembly on nanofibrous and micropatterned polymers. Nano Lett.

[16] Engler AJ, Griffin MA, Sen S, Bönnemann CG, Sweeney HL, Discher DE (2004). Myotubes differentiate optimally on substrates with tissue-like stiffness. J Cell Biol.

[17] Chaturvedi V, Dye DE, Kinnear BF, van Kuppevelt TH, Grounds MD, Coombe DR (2015). Interactions between skeletal muscle myoblasts and their extracellular matrix revealed by a serum free culture system. PLoS One.

[18] Langen RCJ, Schols AMWJ, Kelders MCJM, Wouters EFM, Janssen-Heininger YMW (2003). Enhanced myogenic differentiation by extracellular matrix is regulated at the early stages of myogenesis. In Vitro Cell Dev Biol Anim.

[19] Ahadian S, Ramon-Azcon J, Ostrovidov S, Camci-Unal G, Hosseini V, Kaji H, Ino K, Shiku H, Khademhosseini A, Matsue T (2012). Interdigitated array of Pt electrodes for electrical stimulation and engineering of aligned muscle tissue. Lab Chip.

[20] Altomare L, Gadegaard N, Visai L, Tanzi MC, Farè S (2010). Biodegradable microgrooved polymeric surfaces obtained by photolithography for skeletal muscle cell orientation and myotube development. Acta Biomater.

[21] Lam MT, Huang Y-C, Birla RK, Takayama S (2009). Microfeature guided skeletal muscle tissue engineering for highly organized 3-dimensional free-standing constructs. Biomaterials.

[22] Pennisi CP, Olesen CG, de Zee M, Rasmussen J, Zachar V (2011). Uniaxial cyclic strain drives assembly and differentiation of skeletal myocytes. Tissue Eng A.

[23] Ramon-Azcon J, Ahadian S, Obregon R, Camci-Unal G, Ostrovidov S, Hosseini V, Kaji H, Ino K, Shiku H, Khademhosseini A, Matsue T (2012). Gelatin methacrylate as a promising hydrogel for 3D microscale organization and proliferation of dielectrophoretically patterned cells. Lab Chip.

[24] Strohman RC, Bayne E, Spector D, Obinata T, Micou-Eastwood J, Maniotis A (1990). Myogenesis and histogenesis of skeletal muscle on flexible membranes in vitro. In Vitro Cell Dev Biol.

[25] Wang P-Y, Yu H-T, Tsai W-B (2010). Modulation of alignment and differentiation of skeletal myoblasts by submicron ridges/grooves surface structure. Biotechnol Bioeng.

[26] Bettadapur A, Suh GC, Geisse NA, Wang ER, Hua C, Huber HA, Viscio AA, Kim JY, Strickland JB, McCain ML (2016). Prolonged culture of aligned skeletal myotubes on micromolded gelatin hydrogels. Sci Rep.

[27] Hodge BA, Zhang X, Gutierrez-Monreal MA, Cao Y, Hammers DW, Yao Z, Wolff CA, Du P, Kemler D, Judge AR, Esser KA (2019). MYOD1 functions as a clock amplifier as well as a critical co-factor for downstream circadian gene expression in muscle. eLife.

[28] Liu L, Cheung TH, Charville GW, Rando TA (2015). Isolation of skeletal muscle stem cells by fluorescence-activated cell sorting. Nat Protoc.

[29] Hall DD, Dai S, Tseng P-Y, Malik Z, Nguyen M, Matt L, Schnizler K, Shephard A, Mohapatra DP, Tsuruta F, Dolmetsch RE, Christel CJ, Lee A, Burette A, Weinberg RJ, Hell JW (2013). Competition between α-actinin and Ca2+−calmodulin controls surface retention of the L-type Ca2+ channel CaV1.2. Neuron.

[30] Pasqualin C, Gannier F, Yu A, Malécot CO, Bredeloux P, Maupoil V (2016). SarcOptiM for ImageJ: high-frequency online sarcomere length computing on stimulated cardiomyocytes. Am J Phys Cell Phys.

[31] Bi P, McAnally JR, Shelton JM, Sánchez-Ortiz E, Bassel-Duby R, Olson EN (2018). Fusogenic micropeptide Myomixer is essential for satellite cell fusion and muscle regeneration. Proc Natl Acad Sci.

[32] Feng H-Z, Chen X, Hossain MM, Jin J-P (2012). Toad heart utilizes exclusively slow skeletal muscle troponin T: an evolutionary adaptation with potential functional. J Biol Chem.

[33] Baumann CW, Liu HM, Thompson LV (2016). Denervation-induced activation of the ubiquitin-proteasome system reduces skeletal muscle quantity not quality. PLos One.

[34] Bray NL, Pimentel H, Melsted P, Pachter L (2016). Near-optimal probabilistic RNA-seq quantification. Nat Biotechnol.

[35] Pimentel H, Bray NL, Puente S, Melsted P, Pachter L (2017). Differential analysis of RNA-seq incorporating quantification uncertainty. Nat Methods.

[36] Ashburner M, Ball CA, Blake JA, Botstein D, Butler H, Cherry JM, Davis AP, Dolinski K, Dwight SS, Eppig JT, Harris MA, Hill DP, Issel-Tarver L, Kasarskis A, Lewis S, Matese JC, Richardson JE, Ringwald M, Rubin GM, Sherlock G (2000). Gene ontology: tool for the unification of biology. The Gene Ontology Consortium. Nat Genet.

[37] Consortium, T. G. O (2018). The gene ontology resource: 20 years and still GOing strong. Nucleic Acids Res.

[38] Mi H, Huang X, Muruganujan A, Tang H, Mills C, Kang D, Thomas PD (2016). PANTHER version 11: expanded annotation data from gene ontology and Reactome pathways, and data analysis tool enhancements. Nucleic Acids Res.

[39] Moo EK, Fortuna R, Sibole SC, Abusara Z, Herzog W (2016). In vivo sarcomere lengths and sarcomere elongations are not uniform across an intact muscle. Front Physiol.

[40] Brinegar AE, Xia Z, Loehr JA, Li W, Rodney GG, Cooper TA, Blencowe BJ (2017). Extensive alternative splicing transitions during postnatal skeletal muscle development are required for calcium handling functions. eLife.

[41] Bandyopadhyay A, Dewangan VK, Vajanthri KY, Poddar S, Mahto SK (2018). Easy and affordable method for rapid prototyping of tissue models in vitro using three-dimensional bioprinting. Biocybern Biomed Eng.

[42] Sutherland CJ, Esser KA, Elsom VL, Gordon ML, Hardeman EC (1993). Identification of a program of contractile protein gene expression initiated upon skeletal muscle differentiation. Dev Dyn.

