# Supplementary figures and images for: Culturing C2C12 myotubes on micromolded gelatin hydrogels accelerates myotube maturation
Source: Skelet Muscle. 2019 Jun 7;9:17. doi: 10.1186/s13395-019-0203-4 (PMC6555731; doi:10.1186/s13395-019-0203-4)

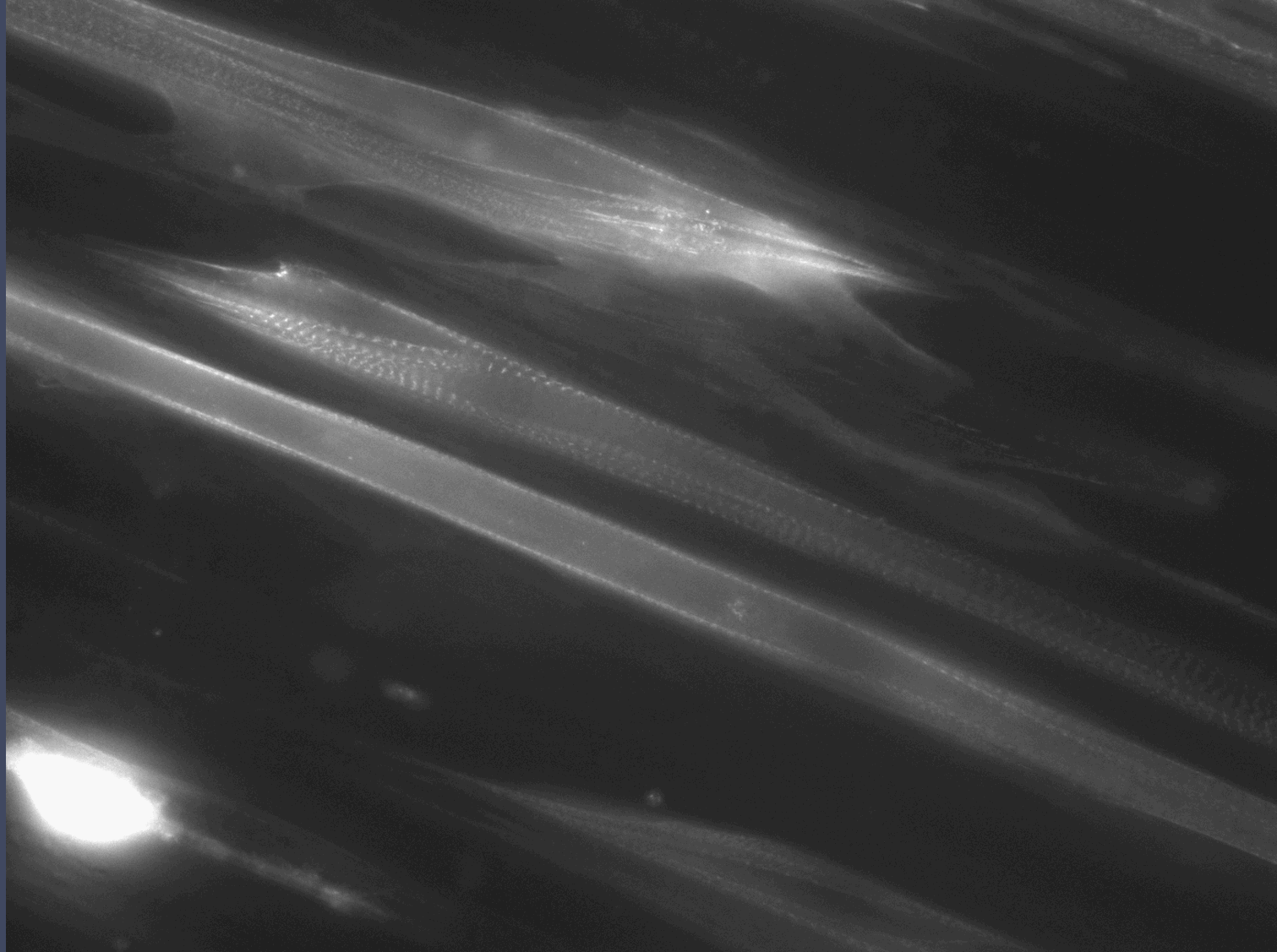

Supplement: Supplementary file 5 — Live cell imaging video of spontaneous contractions in eGFP-a-Actn2 C2C12 myotube on patterned gelatin substrate. (GIF 29121 kb) [file 13395_2019_203_MOESM5_ESM.gif]
